# Biomechanical evaluation over level ground walking of user-specific prosthetic feet designed using the lower leg trajectory error framework

**DOI:** 10.1038/s41598-022-09114-y

**Published:** 2022-03-29

**Authors:** Victor Prost, W. Brett Johnson, Jenny A. Kent, Matthew J. Major, Amos G. Winter

**Affiliations:** 1 Mechanical Engineering, Massachusetts Institute of Technology, Cambridge, 02139 USA; 2 Physical Therapy, University of Nevada, Las Vegas, 89154 USA; 3 Physical Medicine and Rehabilitation, Northwestern University, Chicago, 60611 USA; 4 Jesse Brown VA Medical Center, Chicago, 60612 USA; 5 Biomedical Engineering, Northwestern University, Evanston, 60208 USA

**Keywords:** Mechanical engineering, Musculoskeletal system, Translational research

## Abstract

The walking pattern and comfort of a person with lower limb amputation are determined by the prosthetic foot’s diverse set of mechanical characteristics. However, most design methodologies are iterative and focus on individual parameters, preventing a holistic design of prosthetic feet for a user’s body size and walking preferences. Here we refined and evaluated the lower leg trajectory error (LLTE) framework, a novel quantitative and predictive design methodology that optimizes the mechanical function of a user’s prosthesis to encourage gait dynamics that match their body size and desired walking pattern. Five people with unilateral below-knee amputation walked over-ground at self-selected speeds using an LLTE-optimized foot made of Nylon 6/6, their daily-use foot, and a standardized commercial energy storage and return (ESR) foot. Using the LLTE feet, target able-bodied kinematics and kinetics were replicated to within 5.2% and 13.9%, respectively, 13.5% closer than with the commercial ESR foot. Additionally, energy return and center of mass propulsion work were 46% and 34% greater compared to the other two prostheses, which could lead to reduced walking effort. Similarly, peak limb loading and flexion moment on the intact leg were reduced by an average of 13.1%, lowering risk of long-term injuries. LLTE-feet were preferred over the commercial ESR foot across all users and preferred over the daily-use feet by two participants. These results suggest that the LLTE framework could be used to design customized, high performance ESR prostheses using low-cost Nylon 6/6 material. More studies with large sample size are warranted for further verification.

## Introduction

People with lower limb amputations face considerable challenges to everyday mobility^1^. This impairment impacts about 37 million people globally^2,3^ and affects the quality of life of these individuals due to increased walking effort, social stigmas, and higher incidence of injuries relative to able-bodied individuals^1,4^. To help restore their mobility, people with amputations most commonly use passive prosthetic feet such as energy storage and return (ESR) prostheses, or the more widespread and traditional solid ankle cushioned heel (SACH) prostheses. SACH feet remain the most widely used and distributed prostheses in the world due to their ease of manufacturing, low-cost, and cultural appropriateness in many regions^2,5–7^ (such as biological foot appearance or fully enclosed devices that allow for squatting). Yet ESR prosthetic feet are designed to store and return energy to the user, and have been shown to provide increased benefits and walking performance compared to traditional SACH feet^8–12^. However, ESR prostheses usually cost thousands of US dollars compared to tens of dollars for SACH feet, making them less accessible^7^. This is especially the case in low and middle income countries, which account for 70–80% of the lower limb amputee population^2,3,13^, where the lack of access to affordable, high-performance ESR devices forces people with amputation to use ill-fitted prostheses that reduce their everyday mobility^5^. Creating a user-specific, low-cost, and mass manufacturable ESR prosthetic foot that enables able-bodied walking pattern could significantly improve the access, mobility and quality of life of people with amputation in low and middle income countries.

The current development process of prosthetic feet relies on extensive user testing and iterative design rather than a predictive and quantitative design methodology^14^ that would facilitate the development of improved low-cost, high-performance ESR feet. Enabling able-bodied walking patterns for people with amputation has been one of the goals of prosthetic foot design^15^. More specifically, research on prosthetic foot design has focused on understanding how the mechanical properties of passive prosthetic feet affect the user’s biomechanics^15,16^. These studies have mapped mechanical characterization of prosthetic feet to biomechanical outcomes but mostly demonstrated the effects on locomotion of individual mechanical properties such as stiffness, damping, energy return, and roll-over geometry of a prosthetic foot^10,15^. While these studies provide valuable information on how each mechanical property affects a user’s walking pattern, there is no consensus on how to quantitatively and predictively design a prosthetic foot and tune its mechanical properties to yield a desired biomechanical response^11,14–18^. A foot design methodology that quantitatively connects the entire set of mechanical properties of a prosthetic foot to a user’s biomechanics could be used to develop customized, high performance prostheses, and address specific manufacturing, cost and cultural requirements (such as increased prosthesis compliance and range of motion to enable squatting) to further restore the mobility of people with amputation^5–7,14,15,19^.

**Figure 1:**
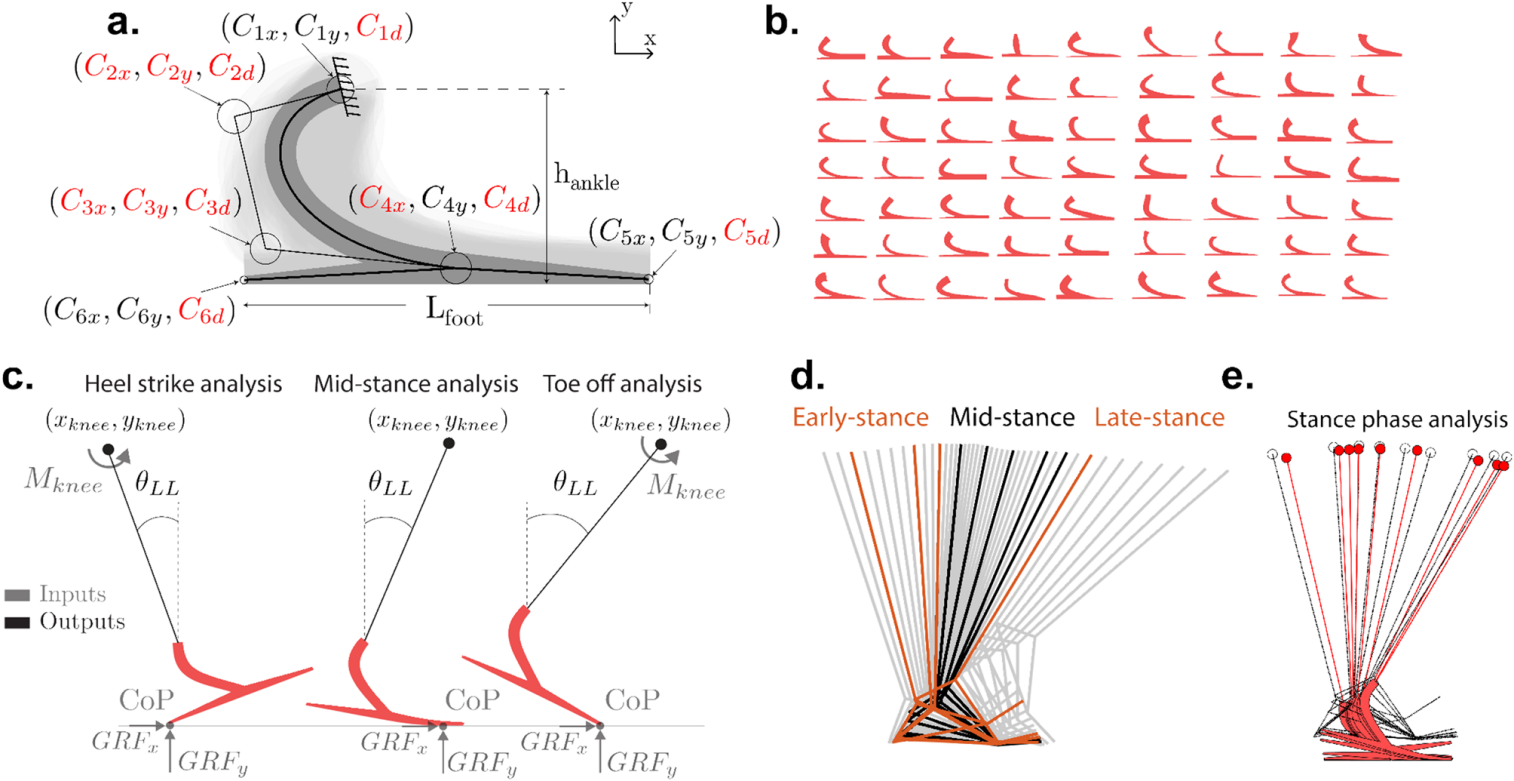
Overview of the LLTE design framework applied in the sagittal plane. (**a**) The prosthetic foot parametric model, shown here overlaid on the foot design space, is defined using the wide Bézier curves’ variables $C_{ij}$, build height $h_{ankle}$, and foot length $L_{foot}$. The design space shown in light grey was created by varying each one of the 11 independent design variables shown in red. (**b**) Sampled prosthetic foot shapes from the design space. (**c**) Prosthetic foot model structural analysis process used to compute the lower leg position and orientation ($x_{knee}$, $y_{knee}$ and $\theta _{shank}$) under a given loading condition (horizontal and vertical ground reaction forces ($GRF_{x}$ and $GRF_{y}$), center of pressure (CoP), and knee moment ($M_{knee}$)). The solid line shows the shank segment connecting the prosthetic foot to the knee joint center. (**d**) Reference gait’s lower leg stance phase trajectory divided into the three main portions of stance (early, mid and late stance), with the selected frames used in the LLTE calculation shown in bold. (**e**) Resulting LLTE-predicted trajectory of the lower leg for a LLTE-optimized prosthetic foot (red) overlaid on the reference trajectory (black).

The lower leg trajectory error (LLTE) framework^20^ is a novel design methodology to deterministically design user-specific prostheses by quantitatively connecting the mechanical characteristics of a prosthetic foot to the gait of an amputee. This methodology enables the systematic tuning of the mechanical properties of passive prosthetic feet (geometry and stiffness) to yield a desired biomechanical response, meet a target cost, and satisfy specific manufacturing requirements^21^. For a given user, a reference kinetic and kinematic walking dataset is scaled to the person’s body characteristics (mass, height and foot length). The LLTE framework then uses a constitutive model of the prosthetic foot to calculate the prosthetic side lower leg trajectory from the deformed prosthetic foot shape when subjected to the target reference walking loads (Fig. 1c). The LLTE is a single value objective that represents the deviation (i.e error) between the calculated prosthetic side lower leg trajectory with that of the target reference lower leg trajectory throughout a step (Eq. 1).

Using the LLTE value as an optimization objective metric, the prosthetic foot’s mechanical characteristics (geometry and stiffness) are then varied to minimize the resulting LLTE value, creating an LLTE-optimal foot design that enables the user to most closely replicate the target walking kinematic and kinetic data. The lower the LLTE value, the closer the replication of the target walking pattern. This LLTE metric has mostly been studied theoretically but has shown promise in an exploratory single participant study as a design objective capable of characterizing the biomechanical behavior of experimental two degree of freedom, jointed prosthetic feet throughout a step^22^, and recent work used the LLTE framework to design low-cost ESR prototypes to replicate a limited portion of a target walking pattern^21^.

In its current form^21^, the LLTE framework step analysis only covers a limited part of a step, where the foot is in dorsiflexion and flat on the ground. It does not include the heel strike nor the late stance portion of a step, resulting in sub-optimal prosthetic foot designs^9,23–25^ that do not have a flexible heel, which led to reported discomfort at heel strike for the users. To further validate the use of the LLTE framework as a deterministic design methodology, the framework has to be extended to design ESR prosthetic feet that replicate the entire target walking pattern through stance phase. In addition, the ability of LLTE optimal ESR prototypes to replicate the target set of walking kinematics and kinetics, and encourage secondary walking benefits such as increased energy storage and return or reduced intact limb loading, should be evaluated against existing devices in a clinical setting.

The aims of this work were to: (1) upgrade the LLTE framework to evaluate prostheses over the entire stance phase and enable the design of ESR prostheses with improved biomechanical function; (2) demonstrate the quantitative and predictive capacity of the LLTE framework to create user-specific, high-performance ESR prosthetic feet without iteration, which closely replicate a target walking pattern; and (3), illustrate that a biomechanically performant ESR prosthetic foot can be designed using low-cost materials with the LLTE framework.

## Upgraded LLTE prosthetic foot design framework

We upgraded the LLTE framework previously defined by Olesnavage et al.^21^ to enable the design of an ESR prosthetic foot architecture with a flexible heel that best replicates the entire target walking pattern through stance. The heel strike and late stance portion of stance were included in the LLTE calculation and a prosthetic foot parametric model with both a flexible heel and keel was developed. The upgraded LLTE framework was then fully implemented in MATLAB (Mathworks, Natick, MA).

### Prosthetic foot parametric model

The prosthetic foot architecture with both a flexible heel and keel was modeled as a 2-D compliant structure using wide Bézier curves^26^ (Fig. 1a). This parametrization was chosen for its simplicity and for producing easily manufacturable designs over traditional topology synthesis methodologies that often include high stress concentration flexural hinges and checkerboard patterns that require extensive post-processing before manufacturing^27–29^. A wide Bézier curve is a parametric shape defined by a series of control points. Using this methodology, a cubic curve can be defined by the position of four control points, reducing a potentially complex shape to a small number of design variables. The thickness of the curve is added as a variable by using control circles rather than control points and defining the thickness of the wide Bézier curve as a function of the diameters of these circles.

Three wide Bézier curves are used to describe this prosthetic foot architecture (Fig. 1a). The main keel portion of the foot is modeled as a cubic wide Bézier curve, using control circles $C_1$ to $C_4$, followed by a linear wide Bézier curve using control circles $C_4$ and $C_5$. The heel portion of the foot is described by a linear wide Bézier curve using the control circles $C_4$ and $C_6$. This foot architecture includes six control circles, each of which are defined by three variables (x-position, y-position and diameter). Of the 18 design variables, 11 independent variables were used in the shape and size optimization, as the remaining 7 were set by the patient’s characteristics (foot length and residuum length) or coupled to an existing variable. The center position of control circle $C_1$ is defined as our reference origin, $C_{5x}$ and $C_{6x}$ are defined by the user’s foot length, and the y-position of circles $C_4$, $C_5$, and $C_6$ are calculated from their circle’s diameter and the foot’s build height, $h_{ank}$. Upper and lower bounds were imposed on each of the independent variables to constrain the shape and size of the structure to approximately fit within the envelope of a biological foot. This parametrization enabled a variety of prosthetic foot designs (Fig. 1b) with varying stiffness and geometry for both the keel and the heel. Each one of the resulting foot designs is a two-dimensional extruded shape that is easily manufacturable with minimal post-processing using waterjet or milling machines, enabling rapid-prototyping and testing.

### Calculation of the LLTE value

For a given prosthetic foot model, the LLTE is calculated by applying the reference walking loads, the ground reaction forces (GRFs), at the specific instantaneous center of pressure (CoP) locations (the location where these loads are applied on the foot) to the prosthesis model through the stance phase (Fig. 1c). From the resulting prosthetic foot deflection, the position and orientation of the lower leg segment ($x_{\mathrm {knee}}$, $y_{\mathrm {knee}}$, and $\theta _{\mathrm {shank}}$) are computed during stance (Fig. 1e). The LLTE value is then calculated as the error between the simulated and reference lower leg trajectory as defined by Olesnavage et al.^20^:

1 $$\begin{aligned} {{\text {LLTE}} = \left[ \frac{1}{N} \sum _{n=1}^N \left\{ \left( \frac{x^{\mathrm {model}}_{\mathrm {knee,n}} - x^{\mathrm {ref}}_{\mathrm {knee,n}}}{\bar{x}^{\mathrm {ref}}_{\mathrm {knee}}} \right) ^2 + \left( \frac{y^{\mathrm {model}}_{\mathrm {knee,n}} - y^{\mathrm {ref}}_{\mathrm {knee,n}}}{\bar{y}^{\mathrm {ref}}_{\mathrm {knee}}} \right) ^2 + \left( \frac{\theta ^{\mathrm {model}}_{\mathrm {LL,n}} -\theta ^{\mathrm {ref}}_{\mathrm {shank,n}}}{\bar{\theta }^{\mathrm {ref}}_{\mathrm {shank}}} \right) ^2 \right\} \right] ^\frac{1}{2},} \end{aligned}$$

where the superscripts “model” and “ref” refer to values calculated by the constitutive model and values from the reference dataset, respectively. N is the total number of frames (time instances of a step) included in the calculation, with n indicating each individual frame. The knee coordinates and lower leg orientation deviations are normalized by the mean of each reference variable across the portion of the step considered (for example, notated by $\bar{x}^{\mathrm {ref}}_{\mathrm {knee}}$ for the knee horizontal coordinate).

Here we extended the calculation of the LLTE metric from the portion of step where the foot is flat on the ground^21^, mid-stance, to the entire step by including the heel-strike and late-stance portions of a step (Fig. 1d). This enables the evaluation of the prosthetic foot’s ability to replicate a target walking pattern over the entire step, which should result in improved LLTE-optimal designs and a more accurate prediction of the prosthetic foot mechanical behavior.

During mid-stance, the position and orientation of the lower leg are fully defined by the position of the CoP along the ground, the applied GRFs, and the prosthetic foot mechanical characteristics, assuming a no-slip condition and the foot to be tangent to the ground at the CoP location^20^ (Fig. 1c). During mid-stance, the prosthetic knee moment can be calculated from the knee coordinates, applied GRFs and CoP locations. A prosthetic foot that closely replicates the reference lower leg trajectory (knee coordinates and lower leg orientation) will thus also replicate the reference knee moment. However, at the portions of stance that immediately follow heel strike and precede toe-off, the prosthetic foot is in line-contact with the ground. During these portions of stance, the entire lower leg system rotates about the stationary center of pressure located at the heel or toe and the angle of the prosthetic foot relative to the ground and the lower leg trajectory cannot be determined from the CoP position and GRF data alone. To resolve the orientation of the lower leg system during heel strike and toe-off, an additional input kinetic data, the knee moment, is required. Applying the reference knee moment data to the simulated lower leg system in addition to the GRFs and CoP positions makes the system fully constrained and allows for the calculation of the foot orientation and thus the entire lower leg position during the point contact instances of the heel strike and toe-off (Fig. 1c). This methodology builds upon the previous LLTE framework process of applying a reference set of kinetic data and calculating the resulting lower leg trajectory for a particular foot model. Details of the equations relating the foot orientation to the CoP position, GRF, and knee moment data can be found in Appendix A. Resolving the foot orientation during heel-strike and toe-off allow for the calculation of the LLTE value of a prosthetic foot over the entire step, improving the performance of LLTE-designed prostheses. With the LLTE value including the heel-strike portion of stance, the prosthetic foot architecture can have both its heel and keel’s geometries and stiffnesses tuned to replicate the target lower leg trajectory during the entire step.

### Application of the LLTE design framework

To design a prosthetic foot using the LLTE framework, a target reference walking dataset that includes both kinematic and kinetic data, and the prosthetic foot material, were first chosen. In our case, a published dataset of able-bodied level ground walking at self-selected speeds from D.A Winter^30^ was selected as the target walking dataset. There is no obvious choice of the target walking dataset to use in the LLTE framework. This able-bodied reference dataset was chosen since our aims for prosthetic foot devices are to restore the biological function of the ankle and enable able-bodied walking patterns. In addition, this reference dataset allowed us to ensure that the experimental foot prototypes and LLTE framework calculations aligned with our previously tested prosthetic feet designed using the LLTE framework^21,22^. The target able-bodied walking data were scaled to the user’s body characteristics, with the GRFs scaled by the user body mass, the CoP locations by the user foot length, and the lower leg trajectory by the user lower leg length. The prosthetic foot height was chosen such that the foot height with the user’s residuum length remains below the user’s lower leg length. Nylon 6/6 was chosen for these prostheses given its low-cost, high strain-energy density ($u \simeq 2.4 \, 10^3$ J/kg), and ease of manufacturing. Its material characteristics were incorporated in the LLTE framework with a tensile modulus $E = 2.51$ GPa, tensile yield stress $\sigma _y = 82.7$ MPa, flexural modulus $E_f = 3.15$ GPa, flexural yield stress $\sigma _{yf} = 92.0$ MPa, poisson ratio $\nu = 0.41$, and density $\rho = 1130$ kg/m$^3$.

Since the LLTE value calculation relies on simulating the deformation of a prosthetic foot model under a set of loading cases using computationally expensive finite element analysis, it was advantageous to minimize the number of stance phase instances included in the LLTE calculation. To determine how many and which instances during the step best represents the step as a whole, the LLTE optimization was applied on simple analytical prosthetic foot models using each combination of stance phase instances, similar to Olesnavage et al.^20^. Here, it was found that using nine loading cases, instances of the stance phase, the LLTE-optimal foot design variable values were each within 5% of the values found using all instances describing stance phase. These representative nine instances were respectively at 8%, 20%, 27%, 36%, 50%, 62%, 75%, 80% and 82% of stance phase, where 0% is heel strike and 100% is toe-off (Fig. 1d). Simulating the prosthetic foot deformation and lower leg trajectory over these nine representative instances of stance phase (Fig. 1e) instead of the 100 instances describing stance resulted in an 10-fold reduction in computational complexity for calculating the LLTE value of a prosthetic foot with a minimal loss in accuracy (less than 5%).

To calculate the LLTE value of a prosthetic foot design, a constitutive structural model of the foot based on 2-D finite frame elements^31^ was implemented in MATLAB. This constitutive model was chosen for its simplicity and reduced computational complexity over commercially available structural analysis software such as ADINA (Watertown, Massachusetts, USA) or SOLIDWORKS (Dassault Systèmes, Vélizy-Villacoublay, France). The prosthetic foot geometry and stiffness described by the 11 independent wide Bézier curves variables was discretized into 300 finite frame elements, determined through a mesh convergence analysis. For each one of the nine loading cases, the prosthetic foot deformations and stress levels were calculated by incrementally applying the GRFs at the corresponding CoP locations along the foot until the structural equilibrium was found. The foot deformations were then used to calculate the prosthetic lower leg trajectory for each instance of stance phase and thus the corresponding LLTE value (Fig. 1e).

The prosthetic foot geometry and stiffness were then varied in order to minimize the prosthetic foot’s LLTE value. This optimization problem was implemented in MATLAB using the built-in genetic algorithm as the optimization function, the 11 independent variables (Fig. 1a) defining the prosthetic foot geometry as the design variables, and the LLTE value as the objective function. A self-intersecting geometric constraint was added to the optimization problem to avoid design variables that result in non-physical structures similar to our previous work^21^. In addition, a stress constraint was included in the optimization problem to ensure that the maximum stress level for all considered loading cases remained below the material’s yield strength with a prescribed safety factor of 1.75 so as to account for unmodelled loading conditions. The integration of the finite element structural model in MATLAB with our LLTE objective function evaluation process and optimization algorithm reduced the need for external software package communication and resulted in an efficient design framework optimization. The LLTE design framework, fully implemented in MATLAB was used to quantitatively design LLTE feet made of low-cost Nylon 6/6, that minimized the LLTE value through the entire stance phase while satisfying the set of stress and geometric constraints using on average one hour of CPU time.

## Methods

### Participants

Five people with a uni-lateral transtibial amputation (70.9 ± 11.1 kg, 1.65 ± 0.05 m, 52.2 ± 8.3 y/o, 15.4 ± 5.1 years post amputation) participated in this study. The sample size was chosen based on previous studies^32–34^ as an initial investigation into this type of research question before conducting larger gait studies. The experimental protocol was approved by the Jesse Brown VA Medical Center Institutional Review Board, (Chicago, IL, USA) and the Committee on the Use of Humans as Experimental participants at the Massachusetts Institute of Technology, (Cambridge, MA, USA). The study and all methods described below were conducted in accordance with the approved protocol. Inclusion criteria included having a uni-lateral transtibial amputation, at least one year of experience walking with a prosthesis, being classified as at least Medicare Functional Classification Level K3, and able to walk continuously for 30 min without undue fatigue or health risks. Exclusion criteria included having a body-mass index greater than 30 and co-morbidities that would affect the intact limb or any pathologies (other than amputation) that might affect balance or stability. All recruited participants fulfilled the inclusion criteria, provided informed written consent prior to data collection and completed the study.

### Prosthetic feet

Three different prosthetic foot conditions were evaluated in this study: an ESR control foot, participants’ daily-use prosthesis, and customized LLTE feet. The Horizon LT foot (College Park Industries, Warren, MI, USA) served as the control prosthesis, as it is a commonly used low-profile, carbon fiber ESR prosthetic foot for moderate to high activity level users, which was unfamiliar to our participants. Each participant also used their daily-use prosthetic foot as a reference comparison, which included a wide range of K3/K4 level carbon fiber ESR prostheses as well as a multi-axial K3/K4 ESR prosthetic foot, listed in Table 1. In addition, all participants used a prototype single part Nylon 6/6 ESR prosthetic foot specifically designed for them using the LLTE design framework described above. For each participant, the resulting LLTE foot design was machined using a waterjet and milling machine, and fitted with a male pyramid adaptor and rubber treads to increase traction (Table 1). The prototype prostheses were all tested under walking loads on a Instron material testing machine (Instron Inc, Norwood, MA) to ensure safety behavior prior to gait analyses. For all prosthetic foot conditions, participants used their own customary prosthetic socket and suspension systems.

**Table 1:** Recruited participants’ characteristics, the corresponding prosthetic foot designed and customized using the LLTE framework, and their daily-use prosthetic foot information.

	Participant 1	Participant 2	Participant 3	Participant 4	Participant 5
LLTE optimal feet geometry					
Manufactured LLTE optimal feet					
LLTE value	0.240	0.465	0.269	0.381	0.398
Age	42	42	58	60	59
Mass	55.9 kg	79.6 kg	61.1 kg	72.5 kg	85.6 kg
Height	1.70 m	1.57 m	1.70 m	1.67 m	1.63 m
Lower leg length	0.440 m	0.454 m	0.505 m	0.498 m	0.459 m
Foot size	0.252 m	0.270 m	0.279 m	0.290 m	0.267 m
Etiology	Traumatic	Vascular	Traumatic	Traumatic	Vascular
Daily-use prosthesis	Ossur Elation	Freedom Innovation ‘Senator’	Fillauer All Pro	Ossur Flex Foot Assure	College Park Trustep

### Experimental protocol

Participants walked over level ground at their self-selected speeds for each prosthetic condition in a randomized order. Over ground walking was chosen to replicate the conditions of the chosen reference data^30^, be more representative of real-world outcomes and limit confounding effects on gait from treadmill protocols^35^. All conditions were tested in a single day session to avoid any inter-day measurement variability^36^ while allowing for as much accommodation and resting time as needed. The LLTE optimal foot was worn without a shoe to most closely match the foot model used in the LLTE framework methodology, while the Horizon LT and daily-use feet were worn with a shoe following the manufacturer’s guidelines. Each participant wore the same model of laboratory-supplied flat shoe (Mossi Damien, Mossimo Supply Co., New York, NY, USA) with the Horizon LT condition to control for and minimize the effect of footwear on stance-phase mechanics^37^. Participants wore their own shoe for the daily-use foot condition so as not to affect the alignment and conditions for their device. The same certified prosthetist performed all prosthetic modifications and clinical (static, dynamic) alignments. Participants were then given as much time to accommodate to the prosthesis until they expressed confidence in the device, before starting the walking trial.

**Figure 2:**
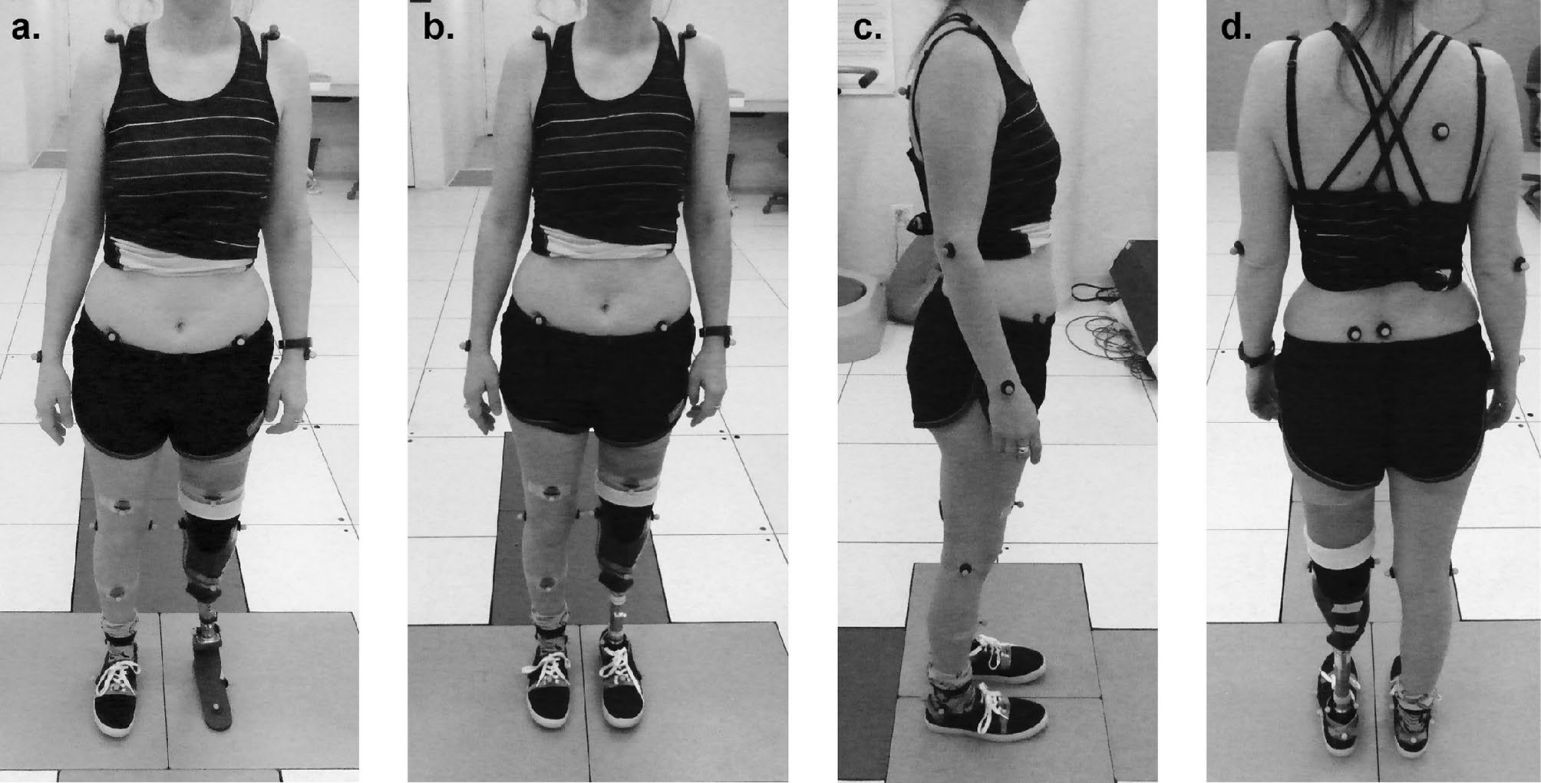
Experimental photographs describing the motion capture marker set placed on a representative participant. Frontal view are shown for the LLTE prosthetic foot condition (**a**) and the Horizon LT control foot condition (**b**). Lateral and back views are shown for the Horizon LT control foot condition (**c**, **d**).

Reflective markers were fixed to anatomical landmarks on the participant by an experienced technician according to a modified Helen Hayes marker set^38^, with markers on the prosthetic foot placed on the pyramid mount, heel, forefoot and toe. Markers on the foot with shoes were placed on the dorsum of the shoe, superficial to the space between the second and third metatarsal heads, and on the heel counter, superficial to the calcaneous, matched in position to the intact foot (Fig. 2). A digital motion capture system (Motion Analysis Corporation, Santa Rosa, CA) collected kinematic data at 120 Hz. Six floor-embedded force plates (Advanced Mechanical Technology, Inc., Watertown, MA) collected kinetic data at 960 Hz. Participants were instructed to walk back and forth along a 10 m walkway at a self-selected comfortable speed. Data from a step were recorded only if the participant’s entire foot landed on a single force plate, and their opposite foot did not contact that same force plate. After at least five steps were collected on each side, the participant’s feedback on the prosthesis was recorded using a prosthetic foot evaluation questionnaire (Supplementary material) before changing the prosthetic foot type. The trial protocol was repeated for each foot condition after a resting, alignment, and acclimation period.

### Biomechanical data analysis

To validate the use of the LLTE framework as a deterministic design methodology, the ability of the LLTE-optimal feet to replicate the target set of walking kinematics and kinetics, and encourage secondary walking benefits such as increased walking speed, energy storage and return, or reduced intact limb loading, was evaluated against an ESR control prosthesis and participants’ daily-use prosthesis, using the collected biomechanical data. A fourth-order bidirectional low-pass Butterworth filter was applied to the kinematic (6 Hz) and kinetic (12 Hz) data to remove noise. Data were then exported to MATLAB to build body segment models. A 40 N vertical GRF threshold detected ground contact and defined the stance phase before calculating the gait variables. Data from each step were represented as percent of the stance phase to account for variations in walking speeds and stance time. For each biomechanical measure, the data were averaged to create a representative step for each participant, foot condition, and for both the prosthetic and intact leg. In addition, data were averaged across participants per leg and foot condition to create group averages after normalizing kinetic data using the participant’s body weight and foot size, and kinematic data using the participant’s lower leg length to account for the participant’s varying body characteristics^39^.

#### Constitutive model validation

During stance phase, the mechanical behavior of the prosthetic foot can be treated as quasi-static^40^ and the prosthetic lower leg motion can be accurately calculated from the prosthetic foot mechanical behavior, GRFs, and CoP data^20–22^. The deterministic mechanical behavior of a lower leg prosthesis does not preclude different users of the same foot to exhibit different gait behaviors; while the constitutive model of the foot remains the same, changes in the loading profile of the prosthesis lead to variations in the prosthetic leg kinematics.

To validate our constitutive model, the position of the knee ($x_{knee}$ and $y_{knee}$) and orientation of the lower leg segment ($\theta _{shank}$) were predicted using the measured GRF and CoP data applied to the mechanical model of the experimental prosthetic foot (Fig. 1). This calculation was similar to those performed in the initial design optimization, but instead of using the published able-bodied data, the kinetic data measured during in-vivo testing from each LLTE feet were used. The lower leg motion during stance predicted by the constitutive model was then compared using a RMSE to the measured lower leg motion from the motion capture system during the single leg support time.

The variables $x_{knee}$, $y_{knee}$, and $\theta _{shank}$ were all defined based on the position of a single “knee” point that, under the assumptions of this analysis, lay on an imaginary vertical line drawn through the ankle joint center when the foot was flat on the ground and unloaded. It was not expected that the knee joint center position, calculated from the lateral knee markers used during data collection, would lie exactly on this vertical line, as each participant’s socket covered the biological knee joint centers making these anatomical features difficult to locate. To account for this discrepancy, a virtual knee marker was defined in post-processing that was the same distance from the ankle as the physical knee marker, but located vertically above the ankle when the foot was on the ground and unloaded. This virtual knee marker was assumed to be part of the same rigid body segment as the physical ankle and knee markers, so the offset angle in the sagittal plane between the virtual knee marker, the ankle marker, and the physical knee marker was kept constant.

#### Deviations from reference walking pattern

The purpose of the LLTE framework is to tune a prosthetic foot’s stiffness and geometry to most closely yield a desired walking pattern (target kinetics and lower leg kinematics). To evaluate how close each prosthetic foot enabled the replication of the target able-bodied walking pattern, the normalized root mean squared error (NRMSE) between the measured and target reference kinematic and kinetic data was computed for both the prosthetic and the intact limb, for each foot tested, over the entire stance phase. The deviations from the target able-bodied walking dataset were grouped into six scores for each leg: GRF deviations (vertical and fore-aft, normalized by body weight), CoP progression deviation (normalized by foot length), and lower leg kinematic deviations ($x_{knee}$, $y_{knee}$ and $\theta _{shank}$ in the sagittal plane as defined in Fig. 1d, normalized by lower leg length and reference $\theta _{shank}$ range, respectively).

In an effort to evaluate the effectiveness of each prosthesis to replicate the reference target biomechanical response, a single deviation score was derived for each participant and for each prosthesis type by summing the kinetic (GRF and CoP) and kinematic (lower leg position and orientation) deviations for both the prosthetic and intact leg. Both legs were considered in this deviation calculation since compensatory motions and loading are usually exhibited on both sides for unilateral amputees^41–44^.

#### Kinematic gait parameters

The following metrics were calculated for each individual step to evaluate the effects of each prosthesis on the participant’s walking dynamics: walking speed, Froude number (Fr)^45^, stance time symmetry index^46^, step width^47^, trunk sway angle^48^, and foot angle^49^. The walking speed was calculated as the average speed over a single trial of the sacrum marker in the direction of travel. To account for the different participant’s body sizes, Froude numbers were calculated as $Fr = v^2/gL$, with *v* the walking speed and *L* the participant’s leg length measured from the hip (greater trochanter) to the floor^45^. The stance phase symmetry index (SI)^46^ was defined as $SI = 100(1-\frac{|X_P - X_S|}{0.5|X_P + X_S|})$, with 100% corresponding to perfect symmetry, and $X_P$ and $X_S$ representing the prosthetic and intact side stance times, respectively. The trunk reference frame was defined using the two shoulder markers and the sacrum marker. The lateral trunk sway angle was then calculated from the trunk reference frame motion in the participant’s frontal plane relative to the lab reference frame^48^. The step width was calculated as the average medial lateral distance between the ankle joint centers at each foot-ground contact^47^.

ESR prosthetic feet are designed to deform in order to store and return energy; the deformation of these compliant structures makes it difficult to define the rotation of the foot segment about the shank segment as a single axis rotation. To overcome this limitation, the prosthetic foot angle was defined as the projection in the sagittal plane of the angle between the foot segment defined by the heel, lateral ankle, and toe markers, and the shank segment defined by the shank, lateral ankle, and lateral knee markers^49^. The foot neutral angle was then calculated during the swing phase of the gait cycle when no forces are applied on the prostheses.

#### Kinetic gait parameters

Roll-over shape radius, effective foot length ratio (EFLR), prosthetic foot power, and step-to-step transition work were calculated in addition to ankle joint moments, foot power, and GRF peak values to evaluate the walking benefits and effects of each prosthesis. The roll-over shape radius is calculated as the radius of the arc described by the CoP in a local reference frame attached to the lower leg from heel strike to the opposite heel strike. The effective foot length is defined as the distance from the heel to the anterior end of the roll-over shape, and corresponds to the location of the CoP at the time of opposite heel contact^40^. The EFLR then corresponds to the ratio of the effective foot length and the physical foot length^50^. The prosthetic foot power was calculated as the distal shank power based on the unified deformable segment model^51^, which treats the foot as a flexible structure and calculates the power absorbed and returned distal to the shank. Since ESR prosthetic feet have no fixed ankle joint axis and violate the rigid body assumption, this methodology may be more appropriate than traditional inverse dynamics calculations^52^. The energy stored and returned by the foot was calculated as the time integral of the foot power during the stance phase of gait and then normalized to body mass. Step-to-step transition work was calculated to define how each limb contributed to the overall propulsion or collision work of the body center of mass (CoM). First, the external mechanical power generated by a limb was computed as the dot product of the limb’s GRF and the velocity of the CoM. Integrating these external mechanical powers during the collision or propulsion phases resulted in the step-to-step transition work^53,54^.

### User feedback

Participant feedback on each prosthetic foot was collected using a prosthetic foot evaluation questionnaire (see Supplementary materials) administered after each walking trial and for each prosthetic condition. This questionnaire captures attributes valued by people with amputation such as comfort, reduced pain, walking effort, stability, confidence or appearance, that are not captured by biomechanical analysis^17,55^ but assisted with results interpretation. Each answer from the evaluation questionnaire was converted into a 5-point likert scale (1-Strongly disagree, to 5-Strongly agree) and summed into a total score out of 50. This prosthesis evaluation score was used to assess the participant’s preference towards a prosthetic foot, with a higher score corresponding to a higher user preference.

### Statistical analysis

All scalar values, such as work and peak force, were first calculated for each step for each individual participant and then averaged across steps and all participants, to avoid any artifacts from averaging. Variance is represented as inter-participant standard deviation for participant averaged data, and as inter-steps standard deviation for individual participant data.

All data were determined to be non-normally distributed via a Shapiro-Wilk test. Therefore, a Friedman’s test was used as a non-parametric, repeated measures analysis of variance to assess group-level main differences between prosthetic foot conditions in all biomechanical variables and participant evaluation scores. Following this, pairwise comparisons were conducted with a Wilcoxon signed test procedure with Holm-Bonferroni corrections to account for family-wise error rates. Statistical analyses were performed in MATLAB with the critical alpha set at 0.05.

Given the small sample size, single-participant analysis was also performed to identify individual responses to prosthetic foot conditions. Using a published MATLAB function^56^ of the Model Statistic tests, a single-participant approach described by Bates^57^ was conducted for the biomechanical variables and the significance level was set for critical $\alpha$ values of 0.05.

## Results

### Constitutive model accurately predicts prosthetic side lower leg motion

**Figure 3:**
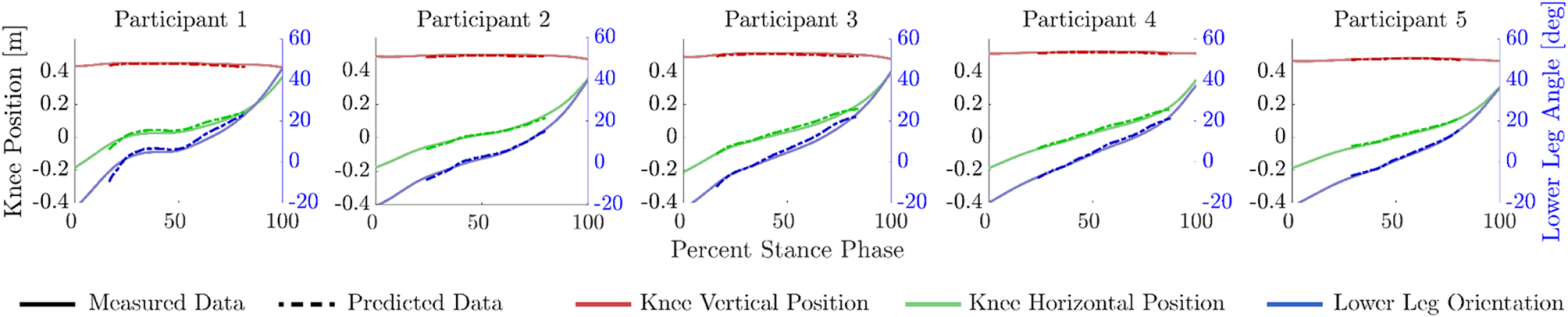
Measured and calculated lower leg trajectories during stance phase for a single representative step for each participant walking with the LLTE prosthesis. The trajectories are defined by the horizontal and vertical coordinates of the knee ($x_{knee}$, $y_{knee}$) and orientation of the lower leg segment ($\theta _{shank}$).

Across five steps per participant using the LLTE feet, the average absolute difference between the predicted and measured lower leg trajectories (Fig. 3) was $1.1 \pm 0.5$ cm for $x_{knee}$, $0.5 \pm 0.2$ cm for $y_{knee}$ and $1.4 \pm 0.8$ deg for $\theta _{shank}$, over a range of motion of $-12$ to 20 cm for $x_{knee}$, and $-15$ deg to 25 deg for $\theta _{shank}$.

### Replication of target kinematics and kinetics

**Figure 4:**
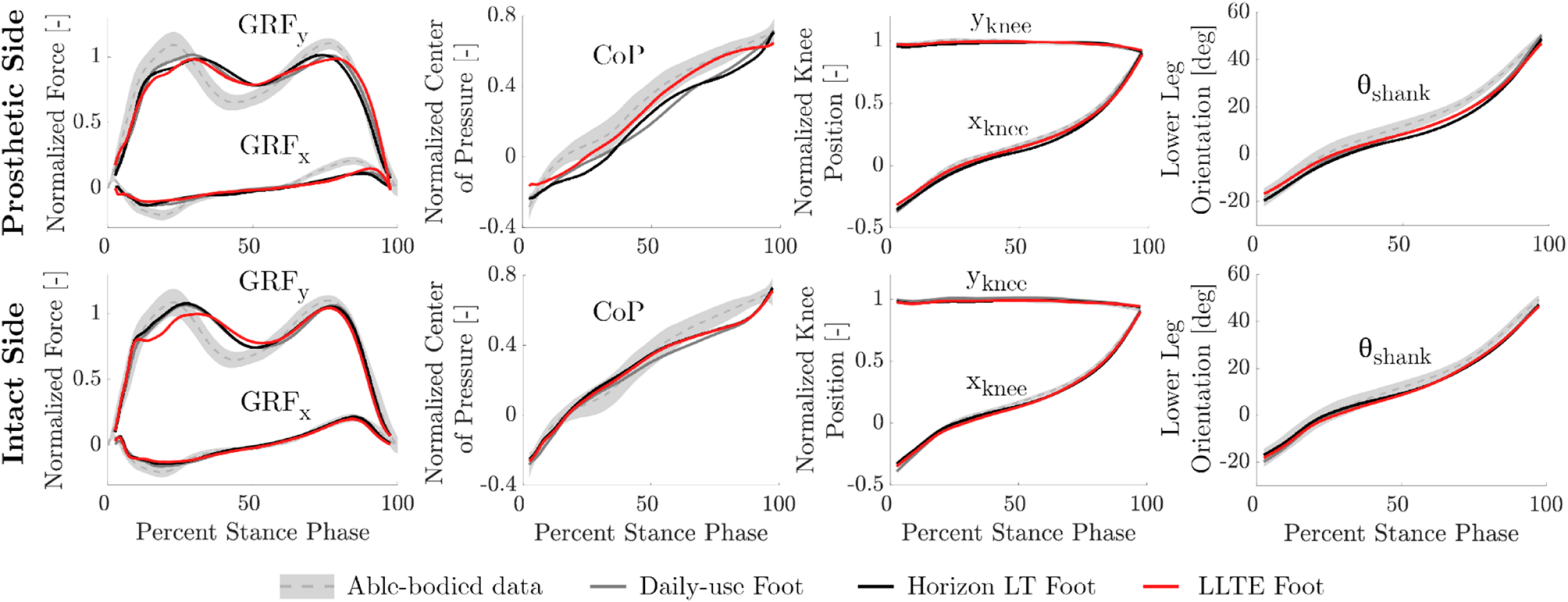
Average kinetic and kinematic variables over the entire stance phase for each prosthetic foot type averaged across all participants. This includes horizontal and vertical ground reaction forces ($GRF_{x}$ and $GRF_{y}$), center of pressure progression (CoP), and lower leg position and orientation in the sagittal plane ($x_{knee}$, $y_{knee}$, and $\theta _{shank}$). Results are shown for both the prosthetic and intact side, and compared to the corresponding reference physiological data^30^ used in the LLTE framework to optimize the feet. The shaded regions correspond to one standard deviation of the normative physiological data.

**Figure 5:**
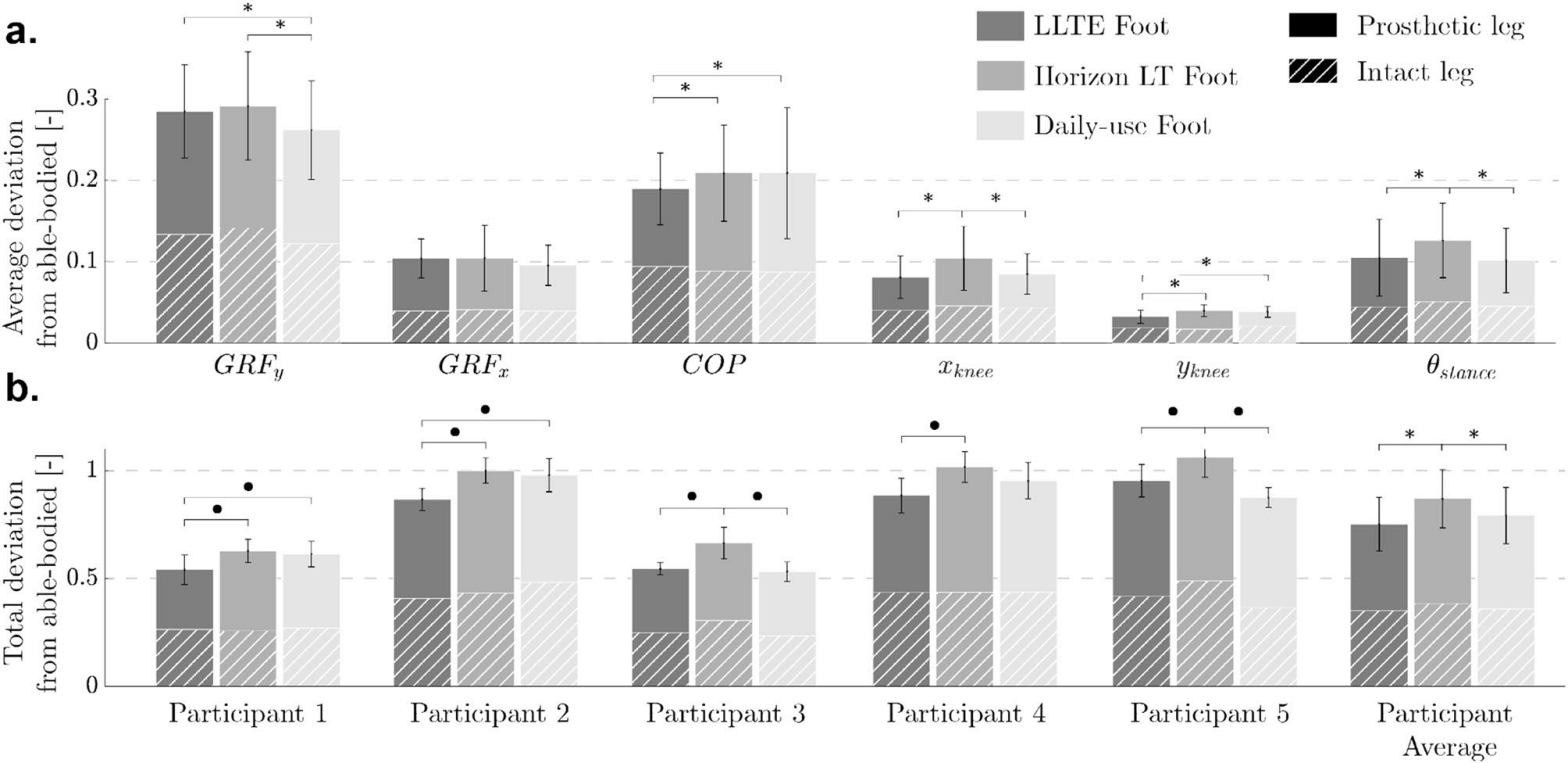
Deviation from able-bodied reference data, calculated using normalized root mean square errors (NRMSE) between the measured walking data and the target able-bodied reference data for each prosthetic foot condition. (**a**) Average deviation for all participants across the different kinematic and kinetic variables. (**b**) Total deviation, summed from all six kinematic and kinetic variables shown for each participant. Group level statistical significance between prosthetic feet conditions is shown with *, and individual statistical significance is shown with •.

LLTE feet, designed quantitatively and predictively, without iteration, and using low-cost Nylon 6/6, replicated the able-bodied lower leg kinematics and kinetics on average within 13.9% for kinetic data and 5.2% for kinematic data (Figs. 4, 5). At a group level, the LLTE feet resulted in 13.5% lower total deviation scores compared to the Horizon LT foot (*p* $< 0.001$) and similar scores compared to the the daily-use foot (*p* = 0.052) (Fig. 5b). This means that LLTE feet enabled participants to replicate the able-bodied target walking kinematics and kinetics closer than with the Horizon LT feet and similarly to the daily-use feet. At the individual level, all participants achieved lower total deviation scores with the LLTE feet than with the Horizon LT foot (Fig. 5b). Two out of the five participants achieved lower deviation scores with the LLTE feet than with their daily-use foot (for participant 1 and 2 (Fig. 5b)). Individual participant’s GRF loading patterns, CoP progression, and lower leg kinematics are provided in Appendix B.

The lower leg kinematics and CoP progression when walking with the LLTE feet were all within one standard deviation of the physiological target (Fig. 4). The GRF loading profiles deviated more than one standard deviation from the target physiological loading pattern around the first vertical GRF peak for both the intact and prosthetic side, and for the horizontal GRF peaks on the prosthetic side. Across all the participants and conditions, most of the deviation from the target physiological walking pattern resulted from the loading pattern (GRFs and CoP), with on average 2.8 times larger deviations compared to the lower leg kinematic data (Figs. 4, 5a). Participants seemed to more closely replicate typical walking motion rather than loading patterns.

### Gait parameters

Regardless of the prosthetic foot type, all participants walked with similar walking speeds, stance time symmetry, step width and trunk sway range of motion (Table 2).

**Table 2:** Main results and gait parameters for the LLTE feet (labeled a), College Park Horizon LT feet (labeled b) and the participant’s daily-use feet (labeled c) while walking over-ground at self-selected speed.

Variables	LLTE$^{\text{a}}$	Horizon LT$^{\text{b}}$	Daily-use$^{\text{c}}$	Effect of foot
Walking speed [m/s]	1.18 ± 0.16	1.18 ± 0.21	1.23 ± 0.13	$p_{ab}$ = 0.307
				$p_{ac}$ = 0.145
Froude number [–]	0.17 ± 0.04	0.17 ± 0.06	0.18 ± 0.03	$p_{ab}$ = 0.319
				$p_{ac}$ = 0.085
Stance time symmetry [–]	94.2 ± 4.8	95.3 ± 3.6	94.0 ± 3.7	$p_{ab}$ = 0.938
				$p_{ac}$ = 0.949
Step width [m]	0.13 ± 0.3	0.13 ± 0.4	0.13 ± 0.3	$p_{ab}$ = 0.979
				$p_{ac}$ = 0.861
Trunk sway range of motion [deg]	6.6 ± 3.6	6.2 ± 3.1	6.3 ± 2.9	$p_{ab}$ = 0.113
				$p_{ac}$ = 0.598
Peak dorsiflexion angle [deg]	11.1 ± 1.4	8.7 ± 0.8*	10.9 ± 4.8	$p_{ab} < 0.001$
				$p_{ac}$ = 0.989
Peak plantarflexion angle [deg]	5.4 ± 0.8	7.7 ± 1.3*	8.9 ± 2.4*	$p_{ab} < 0.001$
				$$p_{ac} < 0.001$$
Roll over shape radius [m/m]	0.33 ± 0.05	0.48 ± 0.14*	0.37 ± 0.07*	$p_{ab}$ = 0.001
				$p_{ac}$ = 0.032
Effective foot length ratio (EFLR) [m/m]	0.78 ± 0.07	0.72 ± 0.05*	0.76 ± 0.05*	$p_{ab} < 0.001$
				$$p_{ac} < 0.001$$
Energy returned by prosthetic foot [J/kg]	0.21 ± 0.08	0.13 ± 0.04*	0.16 ± 0.07*	$p_{ab} < 0.001$
				$$p_{ac} < 0.001$$
Peak prosthetic foot push off power [W/kg]	2.2 ± 0.5	1.2 ± 0.3*	1.7 ± 0.8*	$p_{ab} < 0.001$
				$$p_{ac} < 0.001$$
CoM collision work by prosthetic leg [J/kg]	− 0.05 ± 0.03	− 0.06 ± 0.03	− 0.08 ± 0.03*	$p_{ab}$ = 0.323
				$$p_{ac} < 0.001$$
CoM propulsion work by prosthetic leg [J/kg]	0.18 ± 0.06	0.12 ± 0.03*	0.15 ± 0.05*	$p_{ab} < 0.001$
				$$p_{ac} < 0.001$$
CoM collision work by intact leg [J/kg]	− 0.05 ± 0.03	− 0.10 ± 0.06*	− 0.09 ± 0.05*	$p_{ab} < 0.001$
				$$p_{ac} < 0.001$$
CoM propulsion work by intact leg [J/kg]	0.27 ± 0.05	0.26 ± 0.05	0.26 ± 0.05	$p_{ab}$ = 0.301
				$p_{ac}$ = 0.184
Horizontal GRF second peak on prosthetic leg [N/N]	0.15 ± 0.04	0.11 ± 0.04*	0.12 ± 0.03*	$p_{ab} < 0.001$
				$$p_{ac} < 0.001$$
Vertical GRF first peak on intact leg [N/N]	1.04 ± 0.07	1.16 ± 0.10*	1.11 ± 0.04*	$p_{ab} < 0.001$
				$$p_{ac} < 0.001$$
Intact leg peak knee abduction moment [Nm/kg]	0.26 ± 0.06	0.28 ± 0.05	0.29 ± 0.07*	$p_{ab}$ = 0.109
				$p_{ac}$ = 0.011
Intact leg peak knee flexion moment [Nm/kg]	0.40 ± 0.11	0.49 ± 0.16*	0.53 ± 0.19*	$p_{ab}$ = 0.003
				$p_{ac}$ = 0.002

Values shown here are averaged across all the participants. *p* values between two prosthetic foot condition are shown in the table with the subscript referring to the foot condition’s label. Significant differences to the LLTE feet are denoted by an asterisk *.

#### LLTE feet increased dorsiflexion compared to Horizon LT feet

The LLTE feet enabled increased peak dorsiflexion angle ($p < 0.001$) over the Horizon LT feet but not significantly different from the daily-use feet (*p* = 0.989, Table 2). The LLTE feet led to a reduced peak plantarflexion angle during the loading response at heel strike over the Horizon LT ($p < 0.001$) and daily-use feet ($p < 0.001$). Overall the foot range of motion with the LLTE feet was similar to the Horizon LT feet ($16.5^{\circ }$ and $16.4^{\circ }$, with *p* = 0.882) but smaller than the daily-use feet ($19.8^{\circ }$ with *p* = 0.002).

#### LLTE feet more closely matched physiological roll-over shapes compared to the Horizon LT and daily-use feet

The roll-over shape radius and EFLR were closer to the averaged able-bodied values of 0.31 m/m and 0.81 m/m, respectively^40^, for the LLTE feet compared to both the Horizon LT feet (*p* = 0.001 and $p< 0.001$) and the daily-use feet (*p* = 0.032 and $p < 0.001$, Table 2).

#### Energy return and peak push-off power was greater with the LLTE feet

The LLTE feet demonstrated greater energy return than both the Horizon LT ($p < 0.001$) and daily-use feet ($p < 0.001$) (Table 2). The energy returned by the LLTE feet was 62% and 31% higher compared to the Horizon LT and daily-use feet, respectively. The LLTE feet demonstrated greater peak push-off power than both the Horizon LT ($p < 0.001$) and daily-use feet ($p < 0.001$). The peak push-off power was 83% and 29% higher with the LLTE feet compared to the Horizon LT and daily-use feet, respectively. Individual participant’s and participant average prosthetic foot power are provided in Appendix C.

#### Energy from the LLTE feet impacted whole body center of mass mechanics

The CoM work during propulsion performed by the prosthetic leg was significantly greater with the LLTE feet compared to the Horizon LT and daily-use feet ($p < 0.001$ and $p < 0.001$, respectively, Table 2) which aligns with the trends observed in the previous section describgin prosthesis energy return. There was no significant differences across prosthetic conditions in the CoM work during propulsion performed by the intact leg. The CoM work during collision was lower for the LLTE feet compared to the daily-use feet for both the prosthetic and intact leg ($p < 0.001$ and $p < 0.001$, respectively), but was lower for the LLTE feet compared the Horizon LT feet only for the intact leg ($p < 0.001$) and not the prosthetic leg (*p* = 0.323).

#### LLTE feet led to loading reductions on the intact limb compared to the Horizon LT and daily-use feet

The LLTE feet displayed significantly smaller vertical GRF first peaks on the intact leg compared to the Horizon LT and daily-use feet ($p < 0.001$ and $p < 0.001$, Table 2). The LLTE feet displayed significantly smaller peak knee flexion moment on the intact leg compared to the Horizon LT feet and the daily-use feet (*p* = 0.003 and *p* = 0.002). Similarly, the LLTE feet displayed significantly smaller peak knee abduction moment on the intact leg compared to the daily-use feet (*p* = 0.011) but not significantly smaller compared to the Horizon LT feet (*p* = 0.109) (Table 2).

### User feedback showed participant preference for LLTE feet over Horizon LT feet

**Figure 6:**
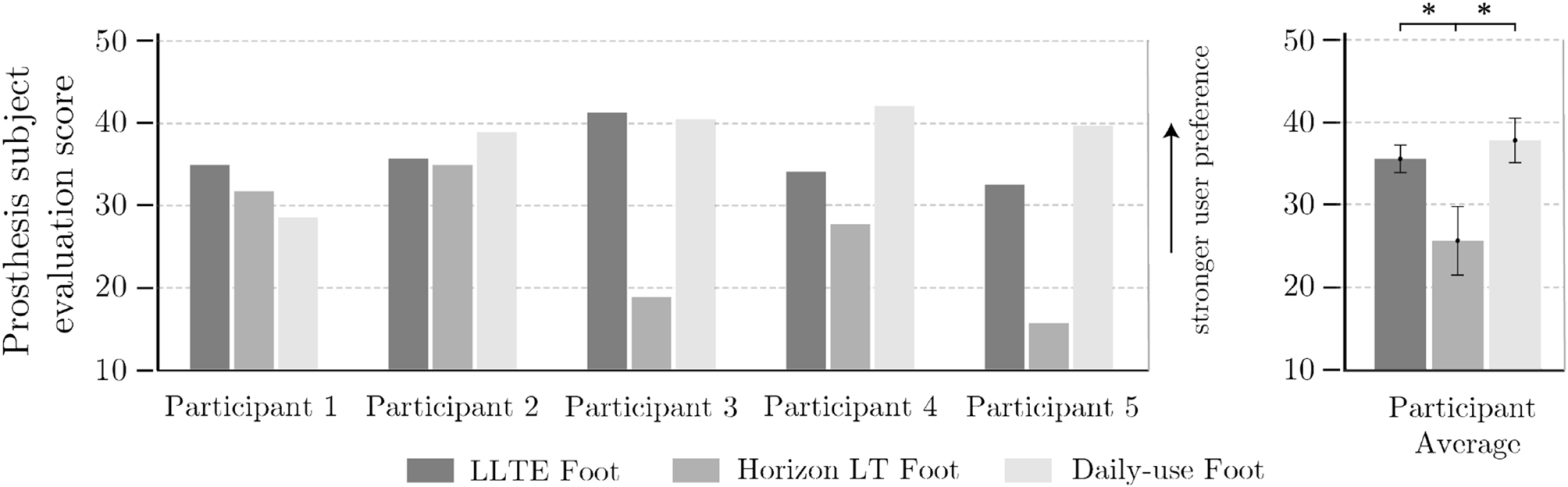
Participants’ prosthesis evaluation scores along with participant-averaged scores for the different prosthetic conditions. Statistical differences are denoted by an asterisk *.

All participants scored the LLTE feet higher than the College Park Horizon LT prosthetic foot, and two participants scored the LLTE foot higher than their daily-use foot (Fig. 6). Across all participants, the LLTE feet and the daily-use feet were perceived better than the College Park Horizon LT feet (*p* = 0.013 and *p* = 0.012), but the difference between the LLTE feet and the daily-use feet was not significant (*p* = 0.195).

## Discussion

The LLTE design framework was developed to quantitatively connect the mechanical characteristics of a prosthetic foot to its anticipated biomechanical performance, in order to design prostheses that yield a desired gait pattern. This study has shown that the upgraded LLTE design framework is a quantitative and predictive design process that produces customized low-cost prostheses which demonstrate closer replication of able-bodied lower leg dynamics (Figs. 4, 5), stronger user preference (Fig. 6), reduced intact limb loading (Table 2), and increased propulsion (Table 2) over a common commercial ESR prosthetic foot (Horizon LT) and similar to the participants’ daily-use feet.

The LLTE framework presented here enabled the analysis of prosthetic feet throughout the entire stance phase, streamlining the design of customized prostheses for specific body size and desired walking activity compared to traditional design processes^14,58^. Integrating the structural analysis with the optimization process within MATLAB and extending the LLTE framework to the entire stance enabled the design of ESR prosthetic feet with both a flexible heel and keel, and improved LLTE values, compared to previous LLTE-optimized prostheses^21,22^. LLTE values of prosthetic feet optimized with the upgraded framework over the entire stance phase were 56% lower than when optimized over only mid-stance, resulting in improved anticipated walking performance over previously designed prostheses^21,22^. The LLTE framework is not restricted to the parametric foot architecture used in this study and could be applied to any prosthetic foot architecture for which a structural model can be built, such as jointed passive prosthetic feet or powered prostheses. In addition, any other material or walking activity could be used when designing LLTE feet by including a reference set of kinematic and kinetic data from these walking activities, such as walking over inclines, or at varying speeds. The upgraded LLTE framework, implemented in MATLAB, enabled the design of customized prostheses with little computational complexity (within an hour of CPU time) compared to computationally intensive simulations^18,59^.

The LLTE framework relies on the prosthetic foot constitutive model to evaluate the LLTE value of the prosthetic foot design. Here we validated that the prosthetic foot constitutive model based on 2D structural frame elements accurately predicted the in vivo prosthetic lower leg trajectory of LLTE feet with an average error of $1.1 \pm 0.5$ cm for $x_{knee}$, $0.5 \pm 0.2$ cm for $y_{knee}$, and $1.4 \pm 0.8$ deg for $\theta _{shank}$ (Fig. 3). The differences between the constitutive model and the measured lower leg trajectories were within the force plate’s accuracy for measuring the CoP locations and GRFs^60,61^, validating the use of 2D frame elements and the prosthetic foot constitutive model.

LLTE feet customized for each participant’s body characteristics had a wide range of foot geometries and stiffnesses specifically optimized using the LLTE framework (Table 1). Despite the varied LLTE foot designs and user’s body characteristics (difference of 30.4 kg in body mass), participants wearing the LLTE feet replicated the reference kinematic variables within 5.2% NRMSE (Fig. 5a) and one standard deviation from the target able-bodied lower leg kinematics (Fig. 4), and kinetic variables within 13.9% NRMSE (Fig. 5a). In addition, across all participants, the LLTE feet provided similar or improved benefits to users compared to commercial ESR prostheses. The results demonstrated that the LLTE feet replicated able-bodied walking patterns significantly better than the Horizon LT foot (16.5% closer replication of the target kinetics and kinematics) and similarly to the daily-use feet across all participants (Figs. 4, 5). The close replication of able-bodied walking patterns with these customized LLTE feet compared to conventional ESR feet supports the use of the LLTE framework as a predictive design tool that quantitatively connects the mechanical characteristics of a prosthetic foot to its biomechanical performance, enabling the design of prostheses that yield a desired gait pattern without iteration.

One assumption behind the LLTE framework is that by designing prostheses that closely replicate able-bodied walking patterns, these devices will be valued by prosthesis users and encourage secondary walking benefits such as increased energy return or reduced intact limb loading^20^. LLTE feet enabled similar walking speeds, stance time symmetry, step width, and trunk sway range of motion as the Horizon LT and daily-use feet. These kinematic gait parameters are usually used as measures of mobility^9^ and stability^62^, suggesting that the LLTE feet might provide similar functionality to the other two prosthetic foot types. In addition, the LLTE optimal prostheses enabled greater propulsion (energy return) than the Horizon LT and the daily-use feet. The difference in energy return between the prostheses cannot be explained by differences in walking speeds. There was no statistically significant differences between all three foot conditions, and at a subject level, the variations in walking speeds were all within $\pm 0.10$ m/s which according to Takahashi et al.^63^ would lead to variations in energy return within $\pm 0.008$ J/kg, almost one order of magnitude below the observed differences in energy return between foot conditions. In addition, positive foot work has been shown to increase with increased walking speed^63–65^. Here, on the contrary, the LLTE feet demonstrated increased energy return compared to daily-use feet while users walked at slower walking speeds, suggesting that the mechanical properties of the prosthetic feet affected the energy return rather than differences in walking speed. This increased propulsion can neither be entirely explained by higher dorsiflexion angles, as has been shown in other studies^41,66^. The LLTE feet achieved higher peak dorsiflexion angles compared to the Horizon LT feet but similar to the daily-use feet, suggesting that the increased energy return is not necessarily linked to increased peak dorsiflexion angles. However, these results also suggest that the LLTE framework can be used to design high energy return prostheses. The progression of the CoP and an increase in EFLR have been shown to increase propulsion^42,67^, but in our case a significant increase in EFLR and closer to able-bodied roll-over shapes were only displayed between the Horizon LT and LLTE feet. The increased push-off power, and returned energy from the LLTE feet led to an increase in CoM propulsion work in the amputated leg compared with the other two prosthetic foot conditions. These increases in propulsion have been shown to reduce the cost of walking^25^, suggesting that LLTE feet could lead to reduced walking effort. Increased propulsion and EFLR in the amputated leg have also shown to reduce loading on the non-amputated leg^24,25,66,68^; here, vertical GRF first peak and peak knee flexion moment were significantly lower when using the LLTE feet compared to the other two feet. Peak knee abduction moments were, however, only significantly lower for the LLTE feet compared to the daily-use feet and not for the Horizon LT feet. These loading reductions in on the non-amputated leg have been shown to reduce the risk of long term injuries such as knee osteoarthritis prevalent among amputees^1,69,70^.

Despite the extensive information provided by gait studies, many attributes valued by prosthesis users can only be captured through prosthesis evaluation questionnaires^17,55^. In this study, the evaluation questionnaire showed that participants preferred the LLTE feet over the Horizon LT and scored it similarly to their daily-use feet. Participants commented on the improved comfort, ‘spring like effect at the toe’, ‘smooth progression from heel to toe’, lightweight, and capacity to ‘walk at a fast pace’ with the LLTE feet compared to the Horizon LT foot. These results suggests that the LLTE feet are valued prostheses that could be adopted by prosthesis users.

The LLTE feet presented in this study were made using low-cost Nylon 6/6 plastic and mass-manufacturable geometries yet were able to provide a closer replication of able-bodied walking patterns, and similar or improved biomechanical performance, compared to conventional carbon fiber ESR prostheses. In addition, these LLTE foot geometries could be over-molded for cosmetic and durability considerations for use in emerging markets, as there are no moving parts. The LLTE design framework could be used to create a design library with a range of prosthetic foot sizes and weight categories, similar to a shoe store with standardized products. Although further testing comparing LLTE feet with SACH feet would be required, these attributes position LLTE feet as a potential alternative to SACH feet and provide access to low-cost, high performance ESR feet that can significantly improve the mobility and quality of life of highly-active amputees in low and middle income countries. In addition, the LLTE framework has the capacity to tune the stiffness and geometry of a prosthetic foot for a specific user’s body characteristics and target walking activity with little computational complexity. Combined with rapid-manufacturing techniques such as CNC machining or additive manufacturing^71,72^, this framework could allow for on-site customized prosthetic foot prescriptions and delivery.

There are several study limitations to consider when interpreting these results. First, the study included a small participant sample size, which limits the generalization of the results demonstrated here to the overall amputee population. Similarly, despite the recruited participants displaying a wide range in body mass and characteristics, the participants were composed of a majority of female amputees (a single male participant), which does not reflect the gender distribution in the overall population. Each participant’s trial was conducted over a single day with little accommodation time, compared to other studies in which participants had multiple sessions or even weeks to acclimatize to a given prosthetic device^73,74^. Additional acclimation time might have resulted in larger variations. Second, the LLTE framework and results described in this study are currently limited to the sagittal plane for simplicity. Although most kinematic behavior and mechanics of level ground walking are constrained to this plane^10,59^, a 3D analysis could provide additional insights into the biomechanics of the three tested foot conditions. Similarly, a 3D optimization methodology could further improve the performance of LLTE designed feet. Third, the Horizon LT and daily-use feet were worn with flat and user-provided shoes that might have affected the biomechanics of the participants^37^ compared to the LLTE prosthetic feet, which were designed to be used without footwear. Lastly, the LLTE prosthetic feet tested in the study were not designed for fatigue performance, and these prostheses did not undergo any of the ISO 10328 or ISO 22765 certification, which should be included in future work.

## Conclusions

The LLTE framework, upgraded to evaluate the LLTE value over the entire stance phase, enabled the quantitative and predictive design of passive prosthetic feet that provide similar or improved benefits compared with traditional carbon fiber ESR prostheses. The LLTE framework was used to design customized ESR prosthetic feet for five prosthesis users to most closely replicate able-bodied level ground walking patterns. The LLTE feet performed as predicted, with no design or fitting iteration required, for a wide variety of patients. This contrasts the iterative and empirical process usually used to design feet, potentially reducing the cost of prosthetic foot development while also providing better-suited prostheses. The LLTE framework presented in this work can be used with any foot architecture, be it active or passive, that can be described by a constitutive model. The LLTE framework was applied on a mass-manufacturable, single part ESR foot architecture using low-cost cost Nylon 6/6. These low-cost, high-performance ESR prostheses could be an alternative to SACH feet especially for highly active users, and significantly improve the mobility and quality of life of amputees in low and middle income countries.

## Supplementary information

Supplementary Information.

## Data Availability

The dataset generated and analysed during the current study are available from the corresponding authors on reasonable request.
