# Supplementary material for: Biomechanical evaluation over level ground walking of user-specific prosthetic feet designed using the lower leg trajectory error framework
Source: Sci Rep. 2022 Mar 29;12:5306. doi: 10.1038/s41598-022-09114-y (PMC8964743; doi:10.1038/s41598-022-09114-y)
Supplement: Supplementary file 1 — Supplementary Information. [file 41598_2022_9114_MOESM1_ESM.pdf]

Supplementary material and appendices

Biomechanical evaluation over level ground walking of user-specific prosthetic feet designed using the Lower Leg Trajectory Error framework

Victor Prost[1,*], W. Brett Johnson[1], Jenny A. Kent[2], Matthew J. Major[3,4,5], and Amos G. Winter V.[1,*]

[1]Massachusetts Institute of Technology, Mechanical Engineering, Cambridge, 02139, United States of America
[2]University of Nevada, Physical Therapy, Las Vegas, 89154, United States of America
[3]Northwestern University, Physical Medicine and Rehabilitation, Chicago, 60611, United States of America
[4]Jesse Brown VA Medical Center, Chicago, 60612, United States of America
[5]Northwestern University, Biomedical Engineering, Evanston, 60208, United States of America
[*]vprost@mit.edu, awinter@mit.edu

SUPPLEMENTARY MATERIAL: Prosthetic foot evaluation questionnaire

The questionnaire used in this study was designed specifically for this protocol to include questions only relevant to high-activity prosthesis users and for single-day prosthetic foot comparison testing. The questions selection and formatting was based on the PEQ-MS (Prosthesis Evaluation Questionnaire-Mobility Subscale)[1] and PLUS-M (Prosthetic limb users survey of mobility)[2].

Subjective Feedback on the Prosthetic Foot

Subject ID:___________ Foot:___________ Date:___________

Please indicate how well you agree with the following statements below.	Strongly Disagree	Disagree	Neutral	Agree	Strongly Agree
1. The prosthetic foot provides a smooth progression from heel to toe:					
2. The prosthetic foot provides enough shock absorption at the heel:					
3. The prosthetic foot provides a spring like effect at the toe end:					
4. The prosthetic foot weighs the right amount:					
5. The prosthetic foot is comfortable to walk on:					
6. The prosthetic foot feels stable to walk on:					
7. I would wear this prosthetic foot for walking at a comfortable pace:					
8. I would wear this prosthetic foot for walking at a fast pace:					
9. I would wear this prosthetic foot for walking in the community:					
10. The prosthetic foot has a nice appearance:					

Please include additional comments on the following topics below.	
11. Please include additional comments on the function of the prosthetic foot.	
12. Please include additional comments on the comfort of the prosthetic foot.	
13. Please include additional comments on the appearance of the prosthetic foot.	

Study Title: Connecting Mechanical to Biomechanical Performance of Prosthetic Feet to Design Customized, Passive Devices that Provide Improved Mobility
IRB Project Number: 115354-1
Version Date: 01/05/2018

Figure S1. Prosthetic foot evaluation questionnaire administered to each participant after each prosthetic foot condition.

APPENDIX

A. LLTE evaluation moment balance

The LLTE framework predicts the prosthetic side lower leg trajectory when subject to a prescribed set of ground reaction forces applied at specific center of pressure locations along the foot. During most part of a step, the prosthetic foot has a portion of its forefoot or heel in contact with the ground[3]. This portion of the prosthetic foot is assumed to be tangent with the ground allowing us to calculate the location of the lower leg from the deformed shape of the prosthetic foot under a given loading case. However, during heel strike and toe-off, the prosthetic foot can be in a line-contact with the ground, being able to freely rotate around the heel or the toe respectively. In these instances, the orientation of the foot and the lower leg, θ_{shank} cannot be resolved solely from the prosthetic foot deformed shape. In these instances of stance, including the reference knee moment in the analysis enables us to calculate the lower leg and foot orientation and fully define the lower leg position. Figure S2 shows the corresponding free-body diagram from which the moment balance around the knee was conducted. Including equations S1 & S2 in the structural analysis of the prosthetic foot deformed shape solves for the lower leg orientation θ_{shank} and allows for the calculation of corresponding prosthetic foot deformation in the ankle reference frame, δ_x and δ_y, at the specific center of pressure location. The lower leg position is then calculated from the lower leg orientation and prosthetic foot deformed shape (Eq. S2)

$$\begin{aligned}\sum M &= 0\\ \sum M &= M_{knee} + GRF_x(y_{knee} - y_{cop}) - GRF_y(x_{knee} - x_{cop})\end{aligned} \tag{S1}$$

$$\begin{aligned}y_{knee} - y_{cop} &= (L_{shank} + h_{ank} - \delta_y)\cos\theta_{shank} + (CoP + \delta_x)\sin\theta_{shank}\\ x_{knee} - x_{cop} &= (L_{shank} + h_{ank} - \delta_y)\sin\theta_{shank} + (CoP + \delta_x)\cos\theta_{shank}\end{aligned} \tag{S2}$$

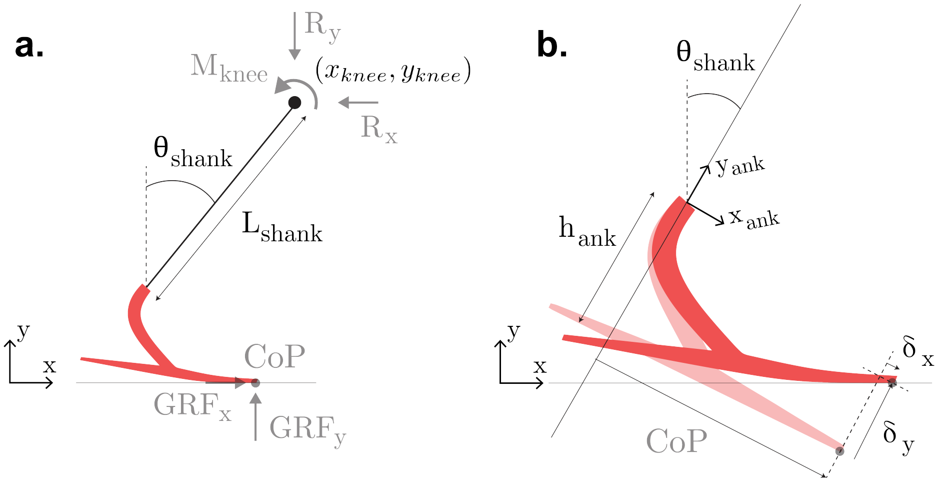

Figure S2. Schematic of the prosthetic lower leg at the end stance with the CoP located at the tip of the prosthesis. a) Free-body diagram of the prosthetic leg with the loads shown in grey and the geomtric dimensions in black. b) Schematic of the prosthetic foot deformations under this loading condition with the shaded red the undeformed shape and the solid red the deformed shape.

B. GRF profiles, CoP and lower leg motion for each participant

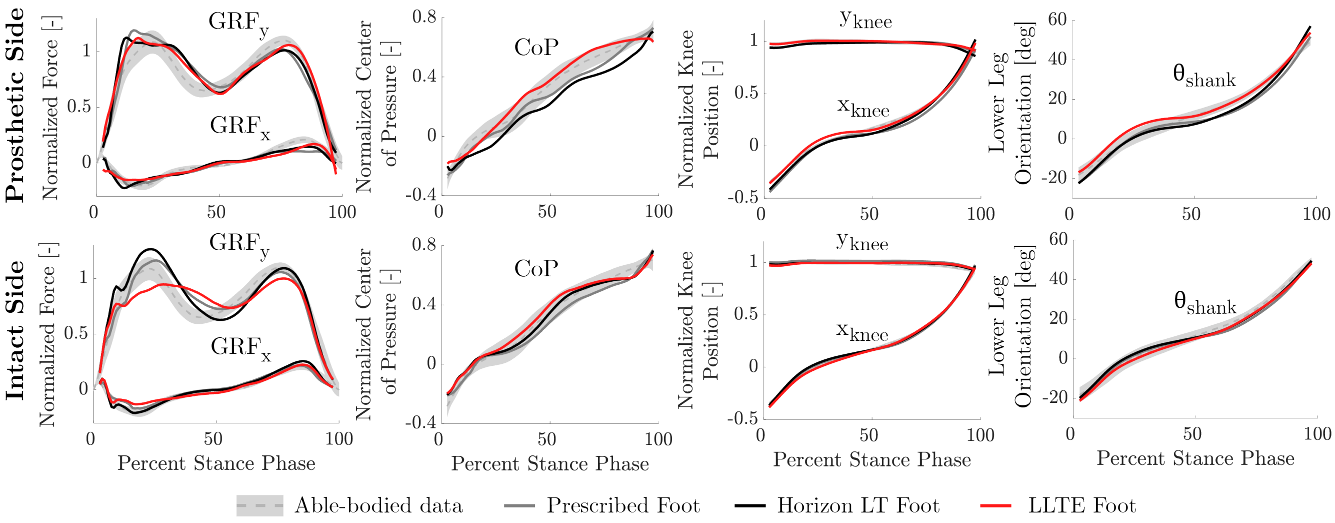

Figure S3. Average kinetic and kinematic variables over the entire stance phase for each prosthetic foot type averaged across all steps for participant 1. This includes horizontal and vertical ground reaction forces (GRF_x and GRF_y), center of pressure progression (CoP), and lower leg position and orientation in the sagittal plane (x_{knee}, y_{knee} and θ_{shank}). Results are shown for the both the prosthetic and intact side, and compared to the corresponding reference physiological data[4] used in the LLTE framework to optimize the foot. The shaded regions correspond to one standard deviation of the normative physiological data.

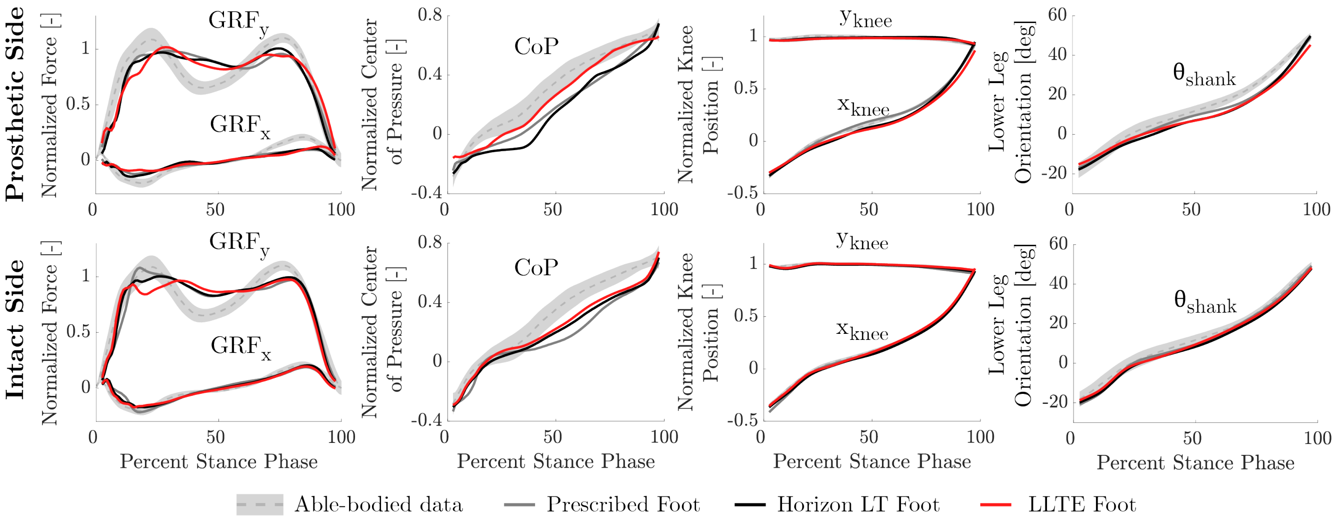

Figure S4. Average kinetic and kinematic variables over the entire stance phase for each prosthetic foot type averaged across all steps for participant 2. This includes horizontal and vertical ground reaction forces (GRF_x and GRF_y), center of pressure progression (CoP), and lower leg position and orientation in the sagittal plane (x_{knee}, y_{knee} and θ_{shank}). Results are shown for the both the prosthetic and intact side, and compared to the corresponding reference physiological data[4] used in the LLTE framework to optimize the foot. The shaded regions correspond to one standard deviation of the normative physiological data.

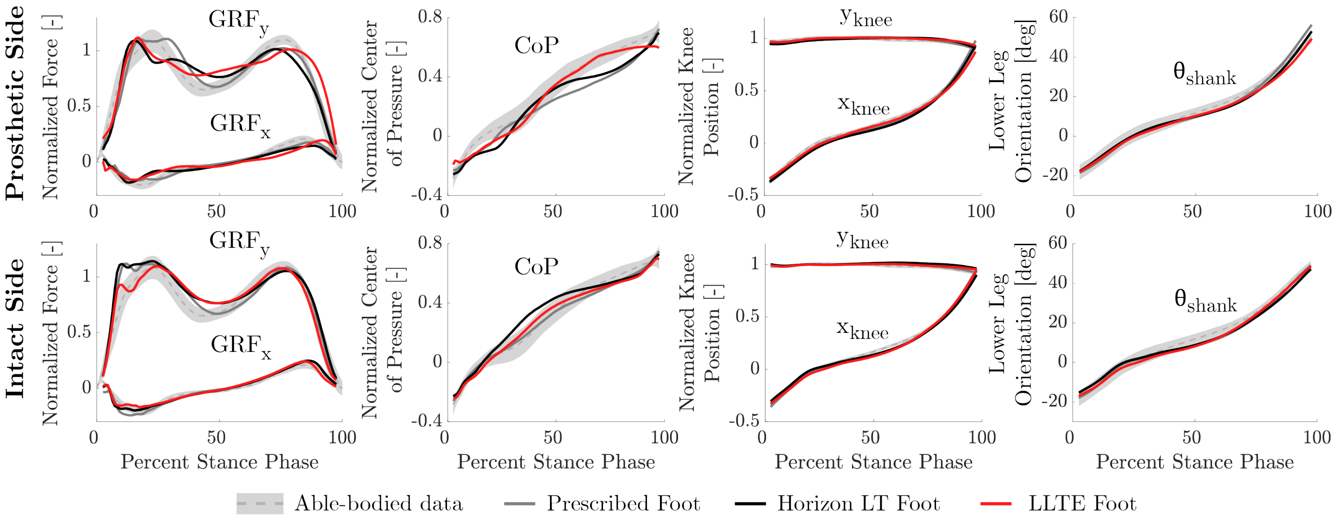

Figure S5. Average kinetic and kinematic variables over the entire stance phase for each prosthetic foot type averaged across all steps for participant 3. This includes horizontal and vertical ground reaction forces (GRF_x and GRF_y), center of pressure progression (CoP), and lower leg position and orientation in the sagittal plane (x_{knee}, y_{knee} and θ_{shank}). Results are shown for the both the prosthetic and intact side, and compared to the corresponding reference physiological data[4] used in the LLTE framework to optimize the foot. The shaded regions correspond to one standard deviation of the normative physiological data.

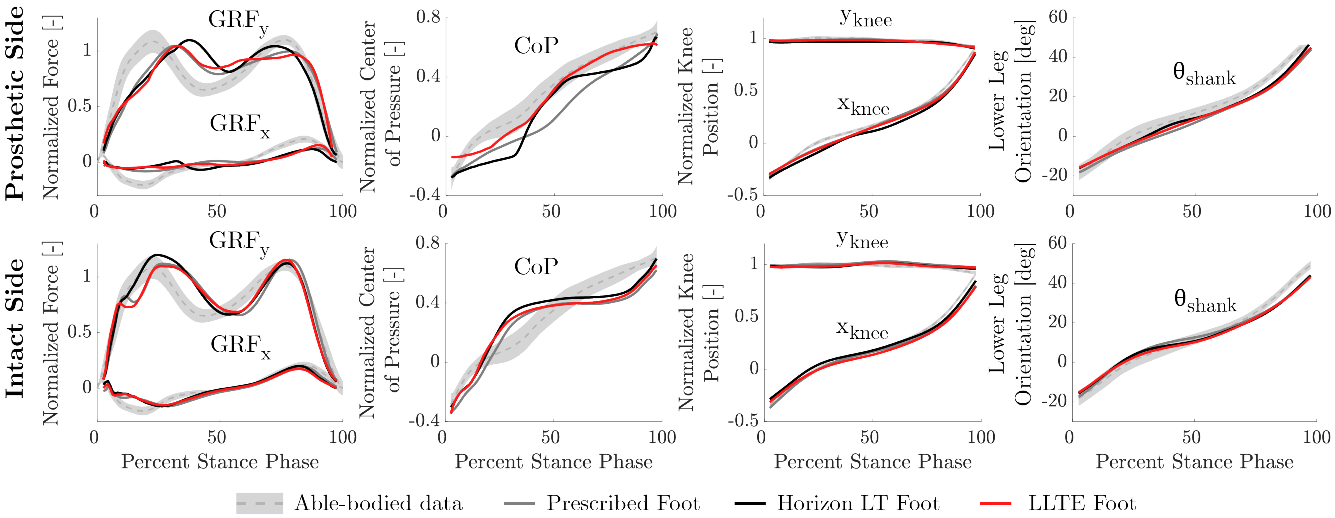

Figure S6. Average kinetic and kinematic variables over the entire stance phase for each prosthetic foot type averaged across all steps for participant 4. This includes horizontal and vertical ground reaction forces (GRF_x and GRF_y), center of pressure progression (CoP), and lower leg position and orientation in the sagittal plane (x_{knee}, y_{knee} and θ_{shank}). Results are shown for the both the prosthetic and intact side, and compared to the corresponding reference physiological data[4] used in the LLTE framework to optimize the foot. The shaded regions correspond to one standard deviation of the normative physiological data.

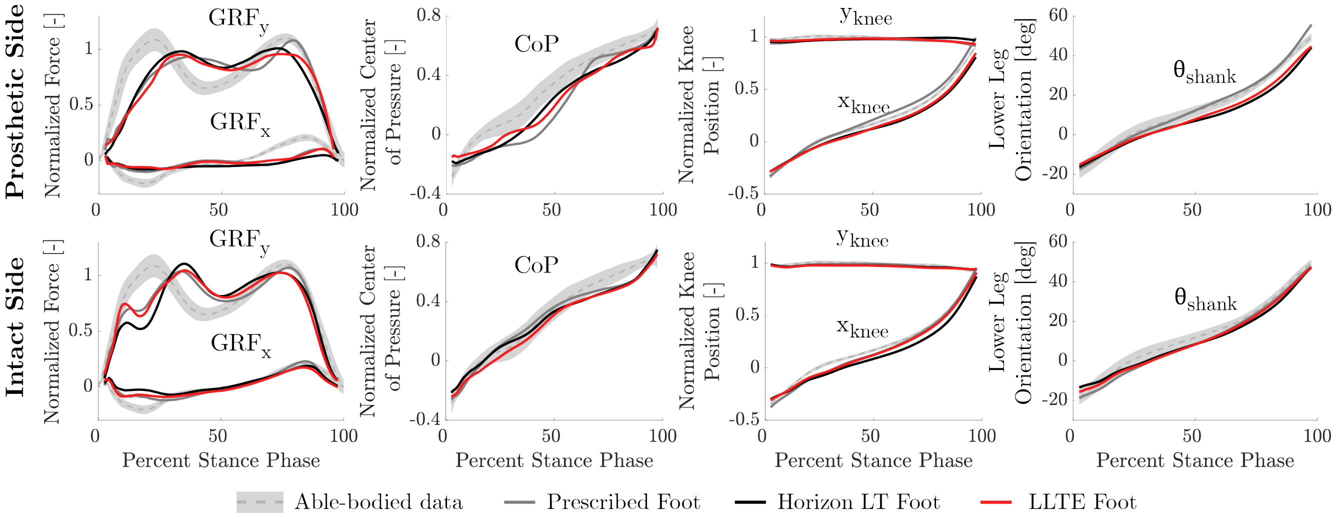

Figure S7. Average kinetic and kinematic variables over the entire stance phase for each prosthetic foot type averaged across all steps for participant 5. This includes horizontal and vertical ground reaction forces (GRF_x and GRF_y), center of pressure progression (CoP), and lower leg position and orientation in the sagittal plane (x_{knee}, y_{knee} and θ_{shank}). Results are shown for the both the prosthetic and intact side, and compared to the corresponding reference physiological data[4] used in the LLTE framework to optimize the foot. The shaded regions correspond to one standard deviation of the normative physiological data.

C. Prosthetic foot power

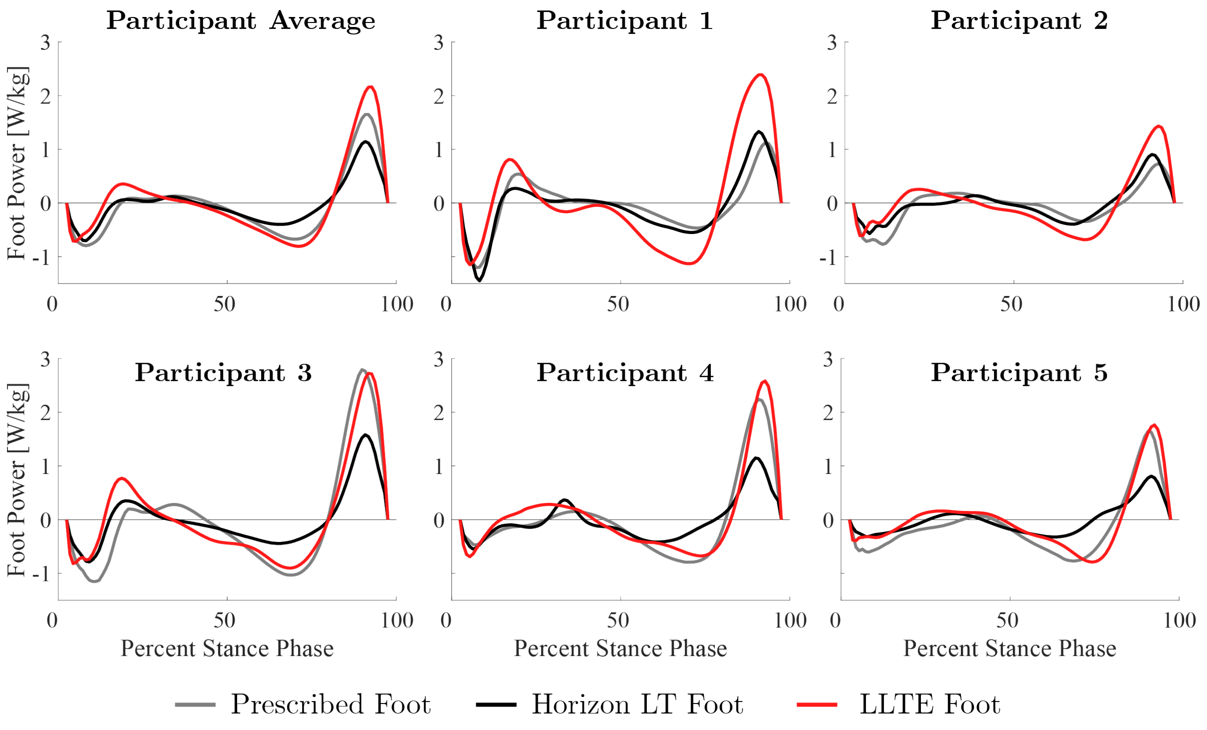

Figure S8. Average prosthetic foot power over the entire stance phase for each prosthetic foot type. The solid horizontal line corresponds to the zero power level.

References

1. Franchignoni, F. *et al.* Measuring mobility in people with lower limb amputation: Rasch analysis of the mobility section of the prosthesis evaluation questionnaire. *J. rehabilitation medicine* **39**, 138–144 (2007).
2. Plus-m™ 12-item short form citation: Prosthetic limb users survey of mobility (plus - m™) 12-item short form (version 1.2). http://www.plus-m.org. Accessed on 2022-01-28.
3. Olesnavage, K. M., Prost, V., Johnson, W. B. & Amos Winter, V. G. Passive prosthetic foot shape and size optimization using lower leg trajectory error. *J. Mech. Des. Transactions ASME* **140**, DOI: 10.1115/1.4040779 (2018).
4. Winter, D. A. *Biomechanics and motor control of human movement* (John Wiley & Sons, 2009).
